# Supplementary material for: Model‐based aberration corrected microscopy inside a glass tube
Source: J Microsc. 2025 Mar 16;298(3):316–23. doi: 10.1111/jmi.13402 (PMC12068014; doi:10.1111/jmi.13402)
Supplement: Supplementary file 1 — Supporting Information [file JMI-298-316-s001.pdf]

Supplementary document

Daniël W.S. Cox, Tom Knop, Ivo M. Vellekoop

Supplementary document for the article *Model-based aberration corrected microscopy inside a glass tube*.

1 Experimental setup

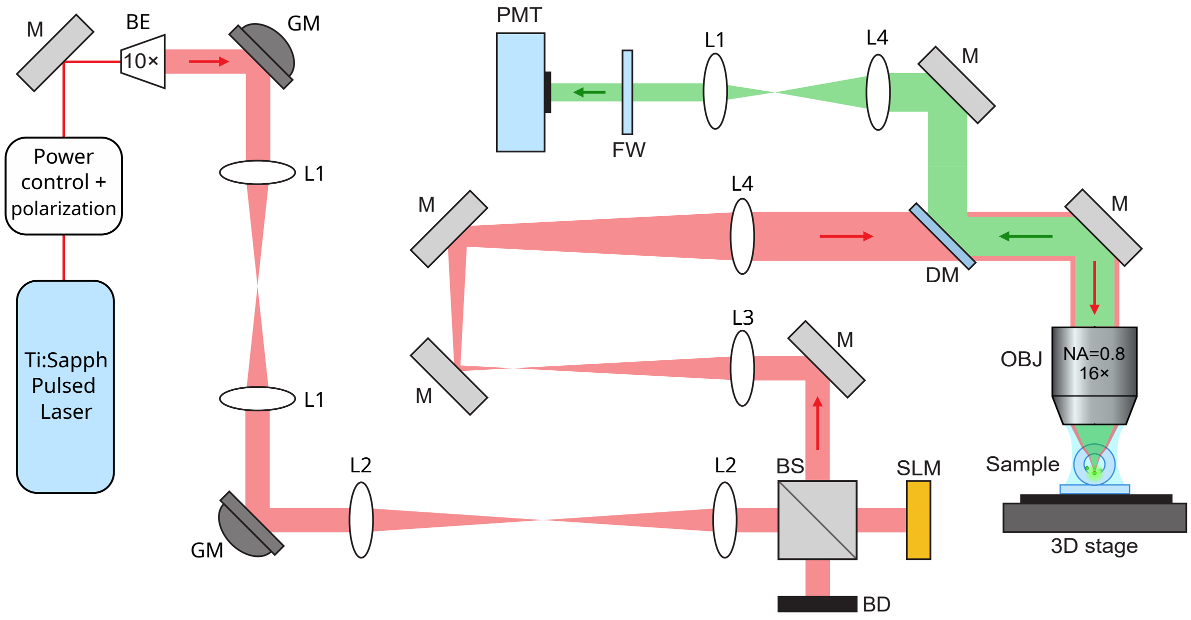

Figure 1: Schematic of the experimental setup. M: mirror, BE: beam expander, GM: galvo mirror, L: lenses with the following focal distances: L1: 100 mm, L2: 200 mm, L3: 150 mm and L4: 300 mm, BS: 50/50 beam splitter, BD: beam dump, SLM: spatial light modulator, DM: dichroic mirror, OBJ: Objective, FW: filter wheel, PMT: Photomultiplier tube. Adapted with permission from [1].

Fig. 1 shows our two-photon excitation fluorescence (2PEF) laser-scanning microscope. We use a titanium-sapphire laser (Spectra-Physics, Mai Tai) as excitation light source at a wavelength of 808 nm. The two galvo mirrors (GM, Thorlabs GVS111/M) are conjugated to the spatial light modulator (SLM, Meadowlark 1920x1152 XY Phase Series), and to the back pupil of the microscope objective (OBJ, Nikon CFI75 LWD 16X W), with 4f-systems. The SLM is illuminated with a polarized beam with a Gaussian amplitude profile. We detect the emitted light with a photomultiplier tube (PMT, Hamamatsu H7422-40).

References

[1] Daniël W. S. Cox and Ivo M. Vellekoop. “Orthonormalization of Phase-Only Basis Functions”. In: *Optics Express* 33.2 (Jan. 2025). `https://opg.optica.org/oe/abstract.cfm?uri=oe-33-2-2427`, pp. 2427–2436. ISSN: 1094-4087. DOI: `10.1364/OE.542622`.
